# Exploring patients’ experience using PROMs within routine post-discharge follow-up assessment after stroke: a mixed methods approach

**DOI:** 10.1186/s41687-024-00724-w

**Published:** 2024-04-26

**Authors:** B.M.P. Mourits, S.J. den Hartog, J.A. de Graaf, B. Roozenbeek, M.W.M. Post, J.M.A. Visser-Meily, E.W.M. Scholten

**Affiliations:** 1 Center of Excellence for Rehabilitation Medicine, UMC Utrecht Brain Center, University Medical Center Utrecht, and De Hoogstraat Rehabilitation, Utrecht, The Netherlands; 2 Department of Neurology, Erasmus MC University Medical Center, Rotterdam, The Netherlands; 3 Department of Rehabilitation, Physical Therapy Science and Sports, UMC Utrecht Brain Center, University Medical Center, Utrecht, The Netherlands; 4 University of Groningen, University Medical Center Groningen, Department of Rehabilitation Medicine, Groningen, The Netherlands

**Keywords:** Patient reported outcome measure, PROM, Stroke, Patient perspectives, Post-discharge consultation, Mixed method, Routine stroke care

## Abstract

**Background:**

Patient Reported Outcomes Measures (PROMs) are being used increasingly to measure health problems in stroke clinical practice. However, the implementation of these PROMs in routine stroke care is still in its infancy. To understand the value of PROMs used in ischemic stroke care, we explored the patients’ experience with PROMs and with the consultation at routine post-discharge follow-up after stroke.

**Methods:**

In this prospective mixed methods study, patients with ischemic stroke completed an evaluation questionnaire about the use of PROMs and about their consultation in two Dutch hospitals. Additionally, telephone interviews were held to gain in-depth information about their experience with PROMs.

**Results:**

In total, 63 patients completed the evaluation questionnaire of which 10 patients were also interviewed. Most patients (82.2–96.6%) found completing the PROMs to be feasible and relevant. Half the patients (49.2–51.6%) considered the PROMs useful for the consultation and most patients (87.3–96.8%) reported the consultation as a positive experience. Completing the PROMs provided 51.6% of the patients with insight into their stroke-related problems. Almost 75% of the patients found the PROMs useful in giving the healthcare provider greater insight, and 60% reported discussing the PROM results during the consultation. Interviewed patients reported the added value of PROMs, particularly when arranging further care, in gaining a broader insight into the problems, and in ensuring all important topics were discussed during the consultation.

**Conclusions:**

Completing PROMs appears to be feasible for patients with stroke attending post-discharge consultation; the vast majority of patients experienced added value for themselves or the healthcare provider. We recommend that healthcare providers discuss the PROM results with their patients to improve the value of PROMs for the patient. This could also improve the willingness to complete PROMs in the future.

## Background

Patients with stroke report limitations in several domains of health [1]. Stroke can lead to physical, psychological, cognitive and social problems that determine whether a patient can resume their daily activities. These problems also impact their perceived health and quality of life [1–4]. While such problems, e.g. fatigue and symptoms of depression, are very common in stroke survivors, they sometimes remain unnoticed during post-discharge follow-up in the outpatient clinic, especially when the problems are not directly visible in physical functioning or behaviour [5–8]. Being aware of the problems experienced by the patient is essential for the healthcare provider to ensure the best-suited care is provided. To help healthcare providers recognise these various problems, Patient Reported Outcome Measurements (PROMs) are being used increasingly during consultations in stroke clinical practice [9–11]. In addition, PROMs can also help involve the patient in the conversation with the healthcare provider and facilitate a discussion of the most relevant problems during the visit [12–14]. Therefore, the use of PROMs supports personalised care by identifying targets for treatment based on patient-reported problems, which could improve the quality of healthcare [9, 15–18].

To understand the value of PROMs during consultations in stroke clinical care, the patient perspective must be involved in their evaluation [19]. In previous quantitative studies, patients were highly satisfied with PROMs used in general neurological practice [20–22]. This satisfaction concerned the understanding and usefulness of the PROM questions, as well as adding value to their visit and perceived care. In addition to the patients’ experiences, rehabilitation physicians are also mostly optimistic about the use of PROMs [23, 24]. However, despite these promising results, the implementation of PROMs more widely in routine stroke care is still in its infancy [25, 26].

In order to increase the usefulness of PROMs in routine post-stroke practice, more in-depth information is required about the patients’ experience with PROMs and about the consultation.

## Methods

### Aim

Our aim was to explore the patients’ experience with PROMs at the first consultation of routine post-discharge follow-up (3–4 weeks) after stroke in two Dutch academic hospitals. By means of a mixed methods approach, we want to answer the following research questions: (1) What is the feasibility and value of PROMs for patients in post-stroke consultations? (2) How do patients experience the consultation with the healthcare provider?

### Study design

In this prospective observational cohort study, we used a mixed methods approach, in which quantitative questionnaire data were complemented with in-depth qualitative data obtained from interviews about how patients experienced the PROMs and the consultation [27]. The Medical Ethics Committee (METC) of Erasmus University Medical Center (Erasmus MC) in Rotterdam declared that this study did not require approval according to the Dutch Law on Medical Research (MEC-2020-0042). The authors report there are no competing interests to declare.

### Study population and setting

The study population consisted of adult patients who had recently suffered ischemic stroke and who had been discharged from hospital. These patients attended a consultation at the outpatient stroke clinic of the Erasmus MC or University Medical Center Utrecht (UMCU) in the Netherlands and they followed the usual outpatient consultation procedure at each hospital (Table 1). Patients were included in the study if they were fluent in the Dutch language and had no severe aphasia, premorbid dementia or psychiatric disorder according to clinical judgement. A specialized nurse and/or rehabilitation physician in outpatient stroke care performed the consultation.

**Table 1:** Protocol for the use of PROMs in routine post-discharge follow-up visits in both participating hospitals

	UMCU	Erasmus MC
Time between admission and visit	6 weeks	3–4 weeks
Healthcare care provider	Neurology nurse specialist and a rehabilitation physician	Neurology nurse specialist
Selection of PROMs	- USER-Participation - EQ-5D+C - PROMIS-10 - HADS	ICHOM outcome set¹ [33]: - PROMIS-10 - Simplified modified Rankin Scale
Location for completing the questionnaire	Home (on paper)	Waiting room or at home (digital)
Contact consultation	In-person, at the outpatient stroke clinic	In-person, at the outpatient stroke clinic

*Abbreviations: PROM* patient reported outcome measure, *USER-Participation* utrecht scale evaluation rehabilitation-participation, *EQ-5D+C* five-dimensional EuroQol+ additional cognitive item, *PROMIS-10* patient reported outcomes measurement information system 10-question short form, *HADS* hospital anxiety and depression scale, *ICHOM* international consortium of health outcomes measurements

¹ICHOM outcomes set is a standard global set of patient reported outcome measures developed for various conditions such as stroke

Both hospitals used their own selection of PROMs, which was already part of usual care. The PROMs measure the following domains: participation (USER-P), health-related quality of life (PROMIS-10 and EuroQoL 5D-5L+C), anxiety and depression symptoms (HADS) and degree of disability (Simplified modified Rankin Scale) [28–32]. Patients complete the PROMs prior to the consultation, so the healthcare providers can use the PROM outcomes as a screening tool during the consultation. The intention is to evaluate the problems a patient experiences after stroke and to indicate whether further treatment is necessary.

Directly after the consultation, we sent a study information letter and an evaluation questionnaire to the patients if they met the inclusion criteria. In the letter they were asked to complete the evaluation questionnaire and that informed consent for the use of the completed questionnaire was approved by returning the questionnaire to the researchers. Informed consent for permission to collect extra data from the patients’ medical record and for a subsequent interview was asked separately at the end of the evaluation questionnaire. A number of patients, who completed the questionnaire and gave consent for the interview, were randomly selected by the researchers for a telephone interview. Inclusion of patients started on 1 March 2020 and ended on 1 June 2022. Because of the COVID restrictions during this period, the researchers temporarily suspended including patients in the study.

### Outcome measurements

We used an evaluation questionnaire to assess the patients’ experience with PROMs and their experience with the consultation. The questionnaire consisted of a selection of 17 statements that have also been used in previous studies evaluating PROMs [22, 34, 35]. All statements were scored on a 5-point Likert scale (strongly disagree to strongly agree). Of all statements, 10 evaluated the patients’ experience of the feasibility and value of PROMs. The other 7 statements evaluated how the patient experienced the consultation with the healthcare provider.

**Fig. 1:**
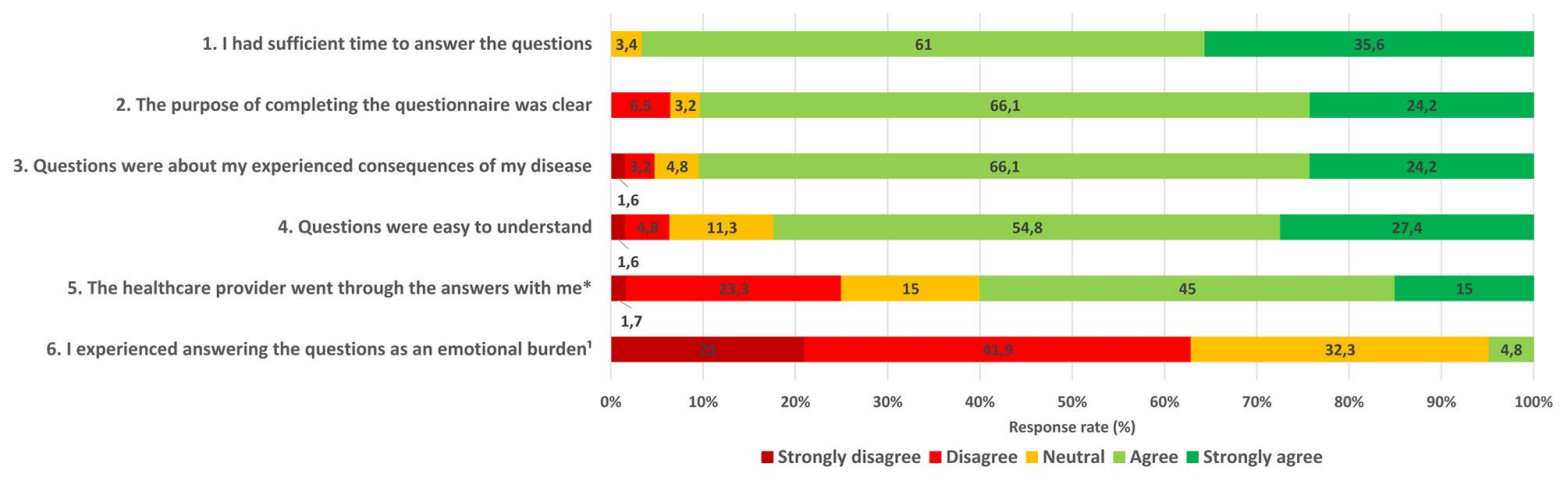
Stroke patients’ experience of the feasibility of PROMs. ^1^In this statement ‘Strongly disagree’ and ‘Disagree’ are labelled as positive answer options. *Statement used in the interview

**Fig. 2:**
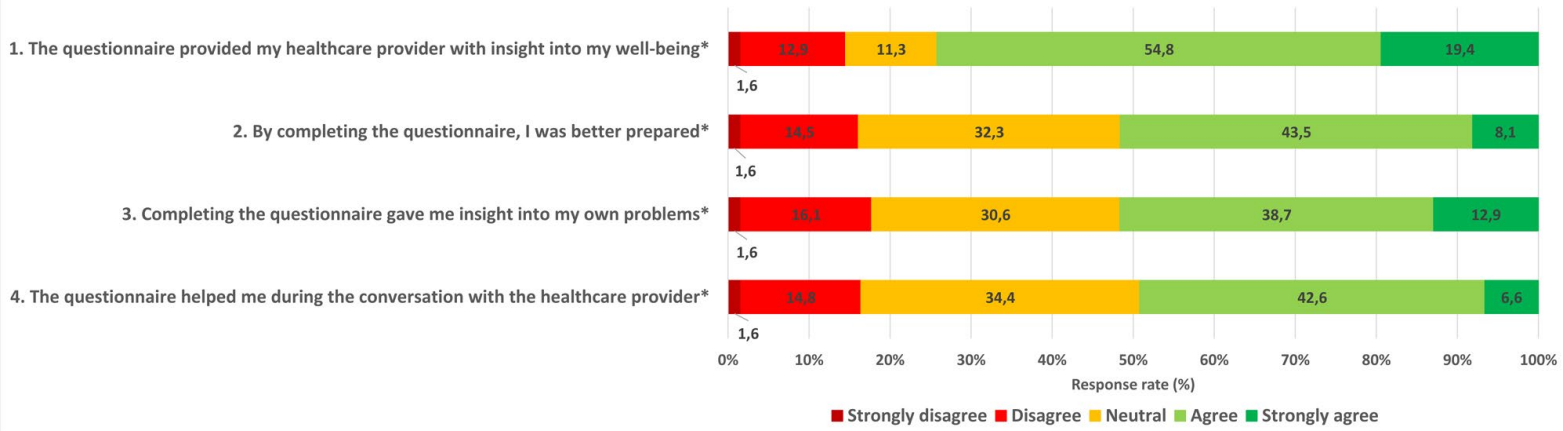
Stroke patients’ experience of the value of PROMs. *Statement used in the interview

We organised a semi-structured telephone interview to gain more detailed information about the patients’ experience with PROMs. The interview consisted of a selection of five statements from the evaluation questionnaire about the value of PROMs (statements with an asterisk in Figs. 1 and 2), each followed by open in-depth questions to clarify their responses to these statements. In addition, two open questions were asked about the added value of completing the PROMs and the comprehensiveness of the PROMs in evaluating all possible problems after stroke. The interviewer (medical student) was affiliated with the UMCU and was not involved in the quantitative data collection. We stopped collecting data when no new information emerged from the last two interviews, i.e. data collection had reached saturation [36].

We collected descriptive information from the patients’ medical record upon admission. Patient characteristics included sex, age, stroke date, date of discharge from hospital, consultation date, location of stroke, Trial of Org 10,172 in Acute Stroke Treatment (TOAST) classification [37], presence of aphasia, National Institutes of Health Stroke Scale (NIHSS) score [38], and Barthel Index [39]. We assessed the TOAST classification to categorize subtypes of stroke based on aetiology. NIHSS and Barthel index were assessed to quantify stroke severity. From the evaluation questionnaire, we retrieved information on education, living situation, self-rated problems with concentration, memory and communication, and also the patients’ preferred location and the time invested in completing the PROMs.

### Analyses

Baseline characteristics and questionnaire data were presented as frequencies and proportions for categorical data, and as mean and standard deviation (SD) or median and interquartile range (IQR) for continuous variables.

The interviewer transcribed the interviews in reports. One researcher (BM) divided the reports into fragments based on the 5 statements and 2 open questions and labelled them with codes: positive response (patient agreed with the statement), neutral response (patient neither agreed nor disagreed with the statement) and negative response (patient disagreed with the statement). Another researcher (ES) independently labelled the fragments of some reports with the codes. Moreover, they looked for the most common clarifications per statement. Quotes were used to support these clarifications. The two researchers (ES and BM) compared the results of coding and common clarifications of some reports and discussed discrepancies.

## Results

In total, 64 patients with an ischemic stroke were included in the study, respectively, 42 patients from the UMCU and 22 patients from the Erasmus MC. We excluded one Erasmus MC patient from the analyses due to absence of consent to use data. This resulted in a total of 63 patients, who gave informed consent and completed the evaluation questionnaire.

Saturation of data collection from the interviews was reached after 11 interviews. Of the 11 interviewed patients, 8 were from the UMCU and 3 from the Erasmus MC. We excluded one interview of an Erasmus MC patient from the analyses. This patient had no active memory of completing the PROMs nor of the consultation and was, therefore, unable to complete the interview. Median time between consultation and interview was 6 weeks (IQR 4–7.25).

More than half of the included patients (54%) were women (Table 2). On admission to hospital, 77% of the patients had mild symptoms resulting from stroke (NIHSS < 5). After the consultation, 14.3% of the patients reported cognitive symptoms (moderate or severe) in the evaluation questionnaire.

**Table 2:** Characteristics of the included patients, stratified by participating hospitals

	Total (n = 63)		UMCU (n = 42)		Erasmus MC (n = 21)	
	n	mean ± SD or median (IQR)	n	mean ± SD or median (IQR)	n	mean ± SD or median (IQR)
**Age (years)**	62	68 ± 1.5	41	70.2 ± 9.1	21	63.6 ± 14.9
**Sex (men)**	61	28 (45.9)	41	20 (48.8)	20	8 (40)
**Location of cerebral infarction**^**a**^**:** Left hemisphere Right hemisphere Cerebellum or brainstem Other	62	22 (35.5) 20 (32.3) 16 (25.8) 4 (6.5)	41	14 (34.1) 15 (36.6) 10 (24.4) 2 (4.9)	21	8 (38.1) 5 (23.8) 6 (28.6) 2 (9.5)
**TOAST classification**^**a**^**:** Large-artery atherosclerosis Cardio embolism Small-vessel occlusion Other and undetermined	62	3 (4.8) 25 (40.3) 15 (24.2) 19 (30.6)	41	3 (7.3) 14 (34.1) 13 (31.7) 11 (26.8)	21	0 (0) 11 (52.4) 2 (9.5) 8 (38.1)
**Aphasiaª (yes)**	61	17 (27.9)	41	11 (26.8)	20	6 (30)
**NIHSSª (0–42)**	44	3 (1.25–4)	23	4 (2–4)	21	3 (0.5–6)
**Barthelª (0–20)**	24	20 (18–20)	10	20 (18.75–20)	14	20 (17.75–20)
**Duration of admission (days)**	62	2 (1–3.25)	41	2 (1–4)	21	2 (1–3)
**Education level**^**b**^**:** Lower education Higher education Other	62	32 (51.6) 25 (40.3) 5 (8.1)	41	19 (46.3) 19 (46.3) 3 (7.3)	21	13 (61.9) 6 (28.6) 2 (9.5)
**Living situation:** Living alone Living with others	63	13 (20.6) 50 (79.4)	42	9 (21.4) 33 (78.6)	21	4 (19) 17 (81)
**Memory**^**c**^**:** No to minor problems Moderate to severe problems	62	55 (88.7) 7 (11.3)	41	38 (92.7) 3 (7.3)	21	17 (81) 4 (19)
**Concentration**^**c**^**:** No to minor problems Moderate to severe problems	63	55 (87.3) 8 (12.7)	42	37 (88.1) 5 (11.9)	21	18 (85.7) 3 (14.3)
**Communication**^**c**^**:** No to minor problems Moderate to severe problems	63	60 (95.2) 3 (4.8)	42	41 (97.6) 1 (2.4)	21	19 (90.5) 2 (9.5)

Categorical variables are presented as percentage n (%). Continuous variables are presented as mean ± SD or median (IQR). *P-values by independent samples t-test and Mann Whitney U test for continuous variables and Chi2 test for binary/categorical variables

*Abbreviations: NIHSS* national institutes of health stroke scale, *TOAST* trial of org 10,172 in acute stroke treatment

^a^Indicated on admission to hospital

^b^Lower education: no completed education, primary school, lower secondary school, higher secondary school or intermediate vocational training; higher education: secondary professional education, completed university or higher

^c^Reported in the evaluation questionnaire

Patients preferred to complete the PROMs at home (100%) and the median time required was 15 min (IQR 10–20). Almost one-quarter (23%) of the patients needed help from others to complete the PROMs.

### Feasibility of PROMs

Most patients agreed with the following statements: ‘*I had sufficient time to answer the questions*’ (96.6%), ‘*The purpose of completing the PROMs was clear*’ (90.3%), ‘*Questions were about my experienced consequences of my disease*’ (90.3%) and ‘*The questions were easy to understand*’ (82.2%) (Fig. 1). Of the patients, 60% agreed with the statement ‘*The healthcare provider went through the answers with me*’. Almost two-thirds of the patients disagreed with the statement *‘I experienced answering the questions as an emotional burden’*.

### Value of PROMs

Three-quarters of the patients agreed with the statement about PROMs being useful for the healthcare providers’ insight (Fig. 2). Half the patients agreed with the statements about PROMs being useful for themselves: better prepared for the consultation (49.6%), provided insight into their problems (51.6%) and helpful during the consultation (49.2%). In addition, approximately 10% of the patients found that PROMs were not useful in providing either them or the healthcare provider with greater insight (disagreed with statements 1 and 3).

The most common clarifications of the interviewed patients with a positive response regarding the statement ‘*By completing the PROMs, I was better prepared*’ were: 1. it gave better insight into the consultation topics and 2. it supported correct expectations about the consultation (Table 3). The most common clarification of a negative response was that the PROMs added little to their own preparation.

**Table 3:** Quotes per statement or interview question

Statements	Positive quotes	Negative quotes
The questionnaire provided my healthcare provider with insight into my well-being	*“It helped enormously with mapping out what still needs to be done in the context of rehabilitation.”*	
	*“It contains questions which you would not ask yourself otherwise or which you do not consider to be important. And of course, they are important for the healthcare provider.”*	
By completing the questionnaires, I was better prepared	*“It helped me, as well as the doctor, to understand what is important to discuss during the consultation.”*	*“No questionnaire would have helped me with the preparation; I myself know what I want to discuss.”*
	*“The questionnaire made it clear what I could expect, and that expectation came true.”*	
Completing the questionnaire gave me insight into my own problems	*“Now you look at the various levels of consequences specifically. This gave a clearer representation of my problems.”*	*“I realized what my problems were before I completed the questionnaire.”* *“I already knew what I was suffering from and what my problems were.”*
	*“I didn’t realise I had a problem. But the questionnaire opened my eyes.”*	
The questionnaire helped me during the conversation with the healthcare provider	*“It gave a nice structure in the conversation and this ensured that issues were not forgotten.”*	*“The conversation with the healthcare provider would have ended the same way even though I did not complete the questionnaires in advance.”*
	*“With the questionnaire, I could express myself well.”*	
**Additional open questions**		
Is it good that a broad range of consequences of stroke are addressed in the PROMs?	*“Otherwise there is a risk that certain matters stay out of sight, but do play a role.”*	
	*“I can imagine that it is very important for people who have more severe consequences from their stroke.”*	
What did you gain from completing the questionnaires?	*“I told the healthcare provider a lot more because of the questionnaire. Including things I would otherwise not have told because I thought it would not be important.”*	*“Personally, the questionnaires did not make me think more about my problems.”*
	*“It gives you a broad representation of how things are going on various levels.”*	*“We said to each other at the consultation that it is good for the doctor to ask these questions.”*

Interviewed patients, who found the PROMs useful during the consultation, gave the following common clarifications: 1. it ensured all topics were discussed and 2. it helped them to express themselves better. Most patients, who found the PROMs useful in gaining insight into their stroke-related problems, gave the clarification that the PROMs helped them achieve a broader perception of the problems. Interviewed patients, who did not find the PROMs useful, mainly reported that they found that PROMs had no added value during their visit nor did they provide insight into their problems.

Almost all interviewed patients responded positively to the statement *‘The questionnaire provided my healthcare provider with insight into my well-being’*, stating that the PROMs provided more insight into the specific care needs of the individual patient, and revealed the topics which were important to the healthcare provider.

Nine out of ten interviewed patients found it was good that the PROMs address a broad range of stroke consequences. Common clarification was that this reduces the risk of missing certain consequences. In addition, three patients clarified that the PROMs are particularly important for patients with severe disabilities.

Regarding the question *‘What did you gain from completing the questionnaires?’*, we summarized the following two benefits: 1. it provided focus on the various problems that are important for themselves and for the healthcare provider and 2. PROMs are useful for gaining insight into possible consequences of stroke. Some patients, who did not benefit from completing the PROMs, still thought they were useful for the healthcare provider.

### Consultation experience

**Fig. 3:**
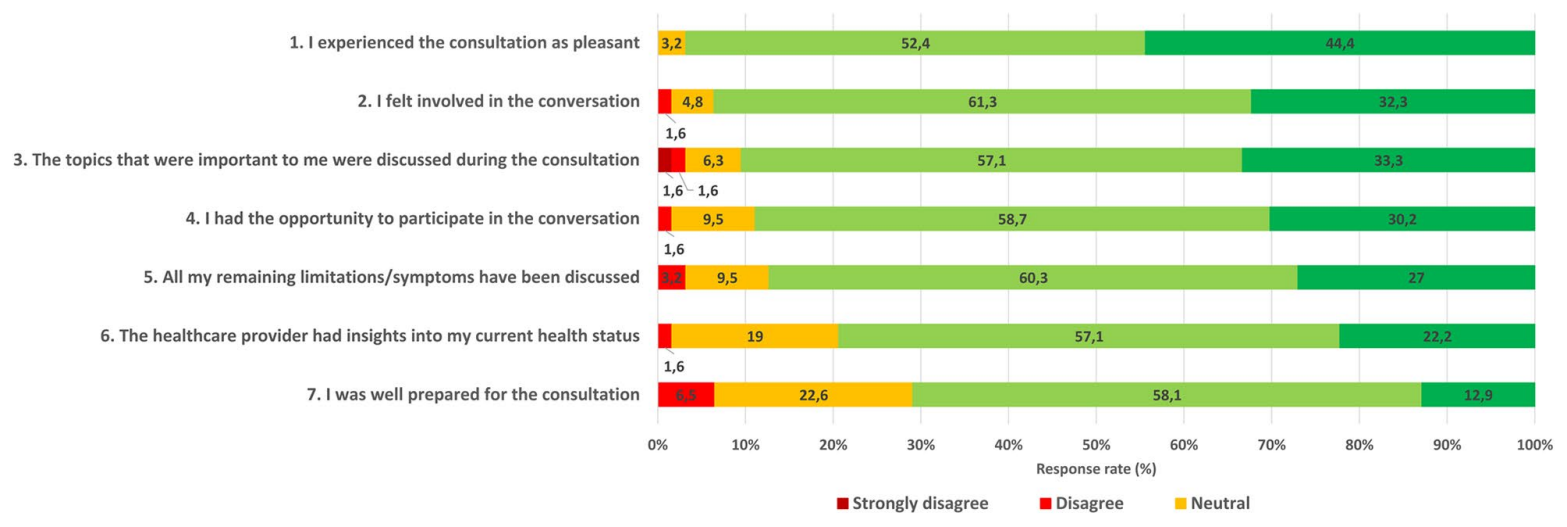
Stroke patients’ experience of the consultation with the healthcare provider

For the vast majority of patients, the consultation with the healthcare provider was a positive experience: patients experienced the conversation as pleasant (96.8%), felt involved (93.6%), agreed that the most important topics were discussed during the visit (90.4%), had the opportunity to participate in the conversation (88.9%), and agreed that remaining limitations were addressed (87.3%) (Fig. 3).

## Discussion

Most patients found completing PROMs to be feasible and effortless. Half the patients found PROMs to be useful and helpful for themselves in preparation for the consultation and during the consultation. Remarkably, more patients found PROMs especially useful for the healthcare provider. For themselves and the healthcare provider, patients reported the added value of PROMs as gaining a broader insight into the problems, ensuring all important topics were discussed, and indicating whether further treatment was needed. Of all patients, only 10% found that the PROMs were not useful in providing greater insight for themselves or the healthcare provider. A large majority of patients were very satisfied with the consultation.

Our results showed that completing PROMs was feasible for patients with stroke. This supports other (feasibility) studies which have revealed that completing PROMs is feasible for patients with neurological disorders and other diseases [20, 22, 23, 40–42]. However, patients with severe neurological impairments were not represented in our study; they may find it more difficult to complete PROMs [9, 43], although another study indicated that feasibility does not differ according to degree of stroke severity [26]. Furthermore, completing PROMs could be challenging for stroke survivors with aphasia, who are often restricted in reading and communication. Completing PROMs with help of another person prior to the consultation could increase the feasibility for these patients and may be of extra added value for them and their healthcare provider in being better prepared to discuss the problems within the limited time of the consultation [44].

Interviewed patients, who found the PROM results useful for the healthcare provider, explained that the PROMs helped the healthcare provider to explore the specific care needs of the patient and provided them insight into topics which were important to discuss. These clarifications are consistent with the purpose of using PROMs in routine healthcare: assisting in patient-centered care and facilitating communication between patient and healthcare provider [45–48]. However, in our study, only 60% of the patients reported that the results of the PROMs were actually discussed during the consultation. Previous studies have revealed that there are various reasons why healthcare providers do not always discuss PROM results with the patient: lack of knowledge about PROMs and their additional value in routine clinical care, restricted time to interpret PROM outcomes and ignorance about how to use PROM data during the consultation [49–52]. It may benefit healthcare providers if they receive some information on how to use and interpret PROMs and also about why it is important to implement them in clinical care [53–57]. We believe that discussing the results of the PROMs is essential, especially when our results showed that patients report more frequently that the PROMs are useful for the healthcare provider than for themselves. Furthermore, if healthcare providers discuss the PROMs during the visit, patients’ perception of the PROMs value and patient adherence to PROM completion are likely to improve [20, 58–60].

The use of PROMs can also help to make routine healthcare more efficient and facilitate patient-centered care [61–65]. When patients with mild stroke symptoms are discharged, 30% of them still experience functional disability [11, 66, 67]. Healthcare providers can then use the PROMs (in combination with other measurements) to screen for these disabilities and use the results to decide whether a follow-up consultation at the outpatient clinic is necessary [68]. If the PROM results indicate that the patient does not experience impactful symptoms or complaints, the healthcare provider can choose to perform the consultation by phone or to skip it completely. If the patient has poor results, the healthcare provider can then choose to perform the consultation at an earlier stage. These options could be cost-effective for both the patient and the healthcare clinics, by preventing unnecessary visits to the hospital, providing appropriate treatment on time and reducing outpatient clinic visits [69]. Evidence supporting these benefits in stroke clinical practice is, however, still lacking; further research is needed [70, 71].

### Limitations

Care pathways across the participating hospitals differed, which led to differences in the selection of PROMs, administrating methods and time points post-admission. A recent study has shown that stroke survivors have certain preferences concerning these aspects of PROM use [72]. Therefore, differences in these aspects could have caused a difference in feasibility between the patients of each hospital. To verify whether this was present in our study, we performed additional exploratory analyses to compare the outcomes of the evaluation questionnaire between the hospitals. This comparison indicated no relevant differences, which could suggest that the differences in procedure had little effect on the patients experience with PROMs.

The study population consisted mainly of patients with relatively mild disabilities after stroke. Although these disabilities were mild, 23% of the patients reported needing help to complete the PROMs. A reason might be because of cognitive or physical problems; however, it might also be due to difficulties with digital devices. People assisting the patient might affect PROM outcomes; however, previous research has shown that there is a moderate to high reliability between patient and proxies for completing PROMs [73]. As in our study a relatively high percentage of proxies was involved in completing the PROMs, we recommend that proxies of patients with stroke are involved in evaluating their use.

In the interviews, some patients reported they had no clear recollection of the consultation or completing the PROMs. Therefore, some patients could not give elaborate answers to the questions; this resulted in less in-depth information. Another qualitative study, in which they used interviews to evaluate PROMs in stroke unit care, reported similar findings [26]. Patients may not remember the visit or completing the PROMs due to cognitive impairments or because of the time period between the consultation and the interview. In our study, the time period was circa 6 weeks. This period was longer than desired, but we could only schedule the interview after we received the evaluation questionnaire, which was sent by mail after the consultation. Therefore the interview could only be scheduled after several weeks. We suggest that in future research, the time between the visit and the interview should be shorter in order to gain more accurate and extensive information.

Finally, we do not know to what extent selection bias, a certain selection of patients who completed the evaluation questionnaire, and social desirability bias, a type of response bias, has occurred in our study. Social desirability bias occurs when persons give answers to questions that they believe will make them look good to others, thus concealing their true opinions or experiences [74, 75]. Although this bias is reduced by patients sending the completed evaluation questionnaire to the research department. To prevent both biases and to strengthen the evidence of the value of PROMs, we suggest performing a study which compares the satisfaction about and experience of the consultation between a group who used PROMs and a group who did not use PROMs.

## Conclusions

Our study results support previous evidence that completing PROMs appears to be feasible for patients in outpatient stroke clinics. The vast majority of patients felt that the PROMs had added value for the healthcare provider, and half the patients for themselves in preparation and during the consultation. Patients reported added values of PROMs as gaining insight into the problems and indicating the need of further treatment by discussing these problems during the consultation. However, some patients reported that the PROM results were not always discussed during the consultation. To improve the value of PROMs for patients, we recommend that healthcare providers discuss the PROM results with the patient during the consultation. We expect that discussing the results will also help improve the willingness of patients to complete PROMs in the future.

## Data Availability

The request to share data of this study will be individually assessed upon reasonable request to the corresponding author.

## List of abbreviations

PROMs: Patient reported outcomes measures
METC: Medical ethics committee
Erasmus MC: Erasmus university medical center
UMCU: University medical center Utrecht
TOAST: Trial of org 10,172 in acute stroke treatment
NIHSS: National institutes of health stroke scale
IQR: Interquartile range
SD: Standard deviation
USER-Participation: Utrecht scale evaluation rehabilitation-participation
EQ-5D+C: Five-dimensional EuroQol + additional cognitive item
PROMIS-10: Patient reported outcomes measurement information system 10-question short form
HADS: Hospital anxiety and depression scale
ICHOM: International consortium of health outcomes measurements

## References

[1] Katzan IL, Thompson NR, Uchino K, Lapin B (2018). The most affected health domains after ischemic stroke. Neurology.

[2] de Graaf JA, van Mierlo ML, Post MWM (2018). Long-term restrictions in participation in stroke survivors under and over 70 years of age. Disabil Rehabil.

[3] Rafsten L, Danielsson A, Sunnerhagen KS (2018). Anxiety after stroke: a systematic review and meta-analysis. J Rehabil Med.

[4] Babulal GM, Huskey TN, Roe CM (2015). Cognitive impairments and mood disruptions negatively impact instrumental activities of daily living performance in the first three months after a first stroke. Top Stroke Rehabil.

[5] Slenders J, Van Den Berg-Vos R, Visser-Meily J (2021). Screening and follow-up care for cognitive and emotional problems after transient ischaemic attack and ischaemic stroke: a national, cross-sectional, online survey among neurologists in the Netherlands. BMJ Open.

[6] Winstein CJ, Stein J, Arena R et al (2016) Guidelines for adult stroke rehabilitation and recovery: a guideline for healthcare professionals from the American Heart Association/American Stroke Association 10.1161/STR.0000000000000098 27145936

[7] Lincoln NB, Brinkmann N, Cunningham S (2013). Anxiety and depression after stroke: a 5 year follow-up. Disabil Rehabil.

[8] Duncan F, Kutlubaev MA, Dennis MS (2012). Fatigue after stroke: a systematic review of associations with impaired physical fitness. Int J Stroke.

[9] Reeves M, Lisabeth L, Williams L (2018). Patient-reported outcome measures (PROMs) for acute stroke: rationale, methods and future directions. Stroke.

[10] Hewitt J, Bains N, Wallis K et al (2021) The use of patient reported outcome measures (Proms) 6 months post-stroke and their association with the national institute of health stroke scale (nihss) on admission to hospital. Geriatr 6. 10.3390/geriatrics6030088 10.3390/geriatrics6030088 PMC8482088 34562989

[11] Sanchez-Gavilan E, Montiel E, Baladas M et al (2022) Added value of patient-reported outcome measures (PROMs) after an acute stroke and early predictors of 90 days PROMs. J Patient-Reported Outcomes 6. 10.1186/s41687-022-00472-9 10.1186/s41687-022-00472-9 PMC9192861 35695977

[12] Aaronson N, Choucair A, Elliott T (2011) User’s guide to implementing patient-reported outcomes assessment in clinical practice. Int Soc Qual Life Res 57 10.1007/s11136-011-0054-x 22048932

[13] Detmar SB, Muller MJ, Schornagel JH (2002). Health-related quality-of-life assessments and patient-physician communication. JAMA.

[14] Valderas JM, Kotzeva A, Espallargues M (2008). The impact of measuring patient-reported outcomes in clinical practice: a systematic review of the literature. Qual Life Res.

[15] Basch E, Spertus J, Adams Dudley R (2015). Methods for developing patient-reported outcome-based performance measures (PRO-PMs). Value Heal.

[16] Chen J, Ou L, Hollis SJ (2013). A systematic review of the impact of routine collection of patient reported outcome measures on patients, providers and health organisations in an oncologic setting. BMC Health Serv Res.

[17] Cella D, Hahn E, Jensen S et al (2015) Patient-reported outcomes in performance measurement 28211667

[18] Bouazza YB, Chiairi I, El Kharbouchi O (2017). Patient-reported outcome measures (PROMs) in the management of lung cancer: a systematic review. Lung Cancer.

[19] Foster A, Croot L, Brazier J (2018). The facilitators and barriers to implementing patient reported outcome measures in organisations delivering health related services: a systematic review of reviews. J Patient-Reported Outcomes.

[20] Recinos PF, Dunphy CJ, Thompson N (2017). Patient satisfaction with collection of patient-reported outcome measures in routine care. Adv Ther.

[21] Lapin BR, Honomichl RD, Thompson NR (2019). Association between patient experience with patient-reported outcome measurements and overall satisfaction with care in neurology. Value Heal.

[22] Lapin B, Udeh B, Bautista JF, Katzan IL (2018). Patient experience with patient-reported outcome measures in neurologic practice. Neurology.

[23] Groeneveld IF, Goossens PH, van Meijeren-pont W (2019). Value-based stroke rehabilitation: feasibility and results of patient-reported outcome measures in the first year after stroke. J Stroke Cerebrovasc Dis.

[24] de Kroon JVV-MA (2013). Klinimetrie: ook nuttig voor individuele patiëntenzorg; toepassing van de USER-P in de dagelijkse praktijk. Ned Tijdschr voor Revalidatiegeneeskunde.

[25] Nic Giolla Easpaig B, Tran Y, Bierbaum M et al (2020) What are the attitudes of health professionals regarding patient reported outcome measures (PROMs) in oncology practice? A mixed-method synthesis of the qualitative evidence. BMC Health Serv Res 20. 10.1186/s12913-020-4939-7 10.1186/s12913-020-4939-7 PMC7011235 32041593

[26] Lebherz L, Fraune E, Thomalla G (2022). Implementability of collecting patient-reported outcome data in stroke unit care – a qualitative study. BMC Health Serv Res.

[27] Fielding NG (2012). Triangulation and mixed methods designs: data integration with new research technologies. J Mix Methods Res.

[28] Post MWM, Van Der Zee CH, Hennink J (2012). Validity of the utrecht scale for evaluation of rehabilitation- participation. Disabil Rehabil.

[29] Feng YS, Kohlmann T, Janssen MF, Buchholz I (2021). Psychometric properties of the EQ-5D-5L: a systematic review of the literature. Qual Life Res.

[30] Lam KH, Kwa VIH (2018). Validity of the PROMIS-10 global health assessed by telephone and on paper in minor stroke and transient ischaemic attack in the Netherlands. BMJ Open.

[31] Spinhoven P, Ormel P, Sloekers PPA (1997). A validation study of the hospital anxiety and depression scale (HADS) in different groups of Dutch subjects. Psychol Med.

[32] Chen X, Li J, Anderson CS (2021). Validation of the simplified modified Rankin scale for stroke trials: experience from the ENCHANTED alteplase-dose arm. Int J Stroke.

[33] Salinas J, Sprinkhuizen SM, Ackerson T (2016). An international standard set of patient-centered outcome measures after stroke. Stroke.

[34] Basch E, Artz D, Dulko D (2005). Patient online self-reporting of toxicity symptoms during chemotherapy. J Clin Oncol.

[35] Snyder CF, Herman JM, White SM (2014). When using patient-reported outcomes in clinical practice, the measure matters: a randomized controlled trial. J Oncol Pract.

[36] Moser A, Korstjens I (2018). Series: practical guidance to qualitative research. Part 3: sampling, data collection and analysis. Eur J Gen Pract.

[37] Adams H, Bendixen B, Kappelle L (1993). Classification of subtype of acute ischemic stroke. Stroke.

[38] Brott T, Adams HP, Olinger CP (1989). Measurements of acute cerebral infarction: a clinical examination scale. Stroke.

[39] Collin C, Wade DT, Davies S, Horne V (1988). The barthel ADL index: a reliability study. Disabil Rehabil.

[40] Gerstl B, Signorelli C, Wakefield CE (2021). Feasibility, acceptability and appropriateness of a reproductive patient reported outcome measure for cancer survivors. PLoS ONE.

[41] Kane PM, Daveson BA, Ryan K (2017). Feasibility and acceptability of a patient-reported outcome intervention in chronic heart failure. BMJ Support Palliat Care.

[42] Franke AD (2021). Feasibility of patient-reported outcome research in acute geriatric medicine: an approach to the “post-hospital syndrome. Age Ageing.

[43] Barrett AM (2009). Rose-colored answers: neuropsychological deficits and patient-reported outcomes after stroke. Behav Neurol.

[44] Hinckley J, Jayes M (2023). Person-centered care for people with aphasia: tools for shared decision-making. Front Rehabil Sci.

[45] Devlin NJ, Appleby J, Buxton M, Vallance-Owen A (2010) Getting the most out of PROMS. Putting health outcomes at the heart of NHS decision making. Health Econ

[46] Churruca K, Pomare C, Ellis LA (2021). Patient-reported outcome measures (PROMs): a review of generic and condition-specific measures and a discussion of trends and issues. Heal Expect.

[47] Nelson EC, Eftimovska E, Lind C (2015). Patient reported outcome measures in practice. BMJ.

[48] Black N (2013) Patient reported outcome measures could help transform healthcare. BMJ 346. 10.1136/bmj.f167 10.1136/bmj.f167 23358487

[49] Graupner C, Breukink SO, Mul S (2021). Patient-reported outcome measures in oncology: a qualitative study of the healthcare professional’s perspective. Support Care Cancer.

[50] Stover AM, Haverman L, van Oers HA (2021). Using an implementation science approach to implement and evaluate patient-reported outcome measures (PROM) initiatives in routine care settings. Qual Life Res.

[51] Greenhalgh J, Abhyankar P, McCluskey S (2013). How do doctors refer to patient-reported outcome measures (PROMS) in oncology consultations?. Qual Life Res.

[52] Briggs MS, Rethman KK, Crookes J (2020). Implementing patient-reported outcome measures in outpatient rehabilitation settings: a systematic review of facilitators and barriers using the consolidated framework for implementation research. Arch Phys Med Rehabil.

[53] Der Willik EMV, Milders J, Bart JAJ (2022). Discussing results of patient-reported outcome measures (PROMs) between patients and healthcare professionals in routine dialysis care: a qualitative study. BMJ Open.

[54] Antunes A, Racha-Pacheco R, Esteves C et al (2023) PRO-act: a healthcare provider workshop outlining the added value of implementing PROs in routine HIV practice. J Patient-Reported Outcomes 7. 10.1186/s41687-023-00584-w 10.1186/s41687-023-00584-w PMC10235267 37261556

[55] Santana MJ, Haverman L, Absolom K (2015). Training clinicians in how to use patient-reported outcome measures in routine clinical practice. Qual Life Res.

[56] Brunelli C, Zito E, Alfieri S (2022). Knowledge, use and attitudes of healthcare professionals towards patient-reported outcome measures (PROMs) at a comprehensive cancer center. BMC Cancer.

[57] Campbell R, Ju A, King MT, Rutherford C (2022). Perceived benefits and limitations of using patient-reported outcome measures in clinical practice with individual patients: a systematic review of qualitative studies. Qual Life Res.

[58] Velikova G, Keding A, Harley C (2010). Patients report improvements in continuity of care when quality of life assessments are used routinely in oncology practice: secondary outcomes of a randomised controlled trial. Eur J Cancer.

[59] Lombi L, Alfieri S, Brunelli C (2023). ‘Why should I fill out this questionnaire?’ A qualitative study of cancer patients’ perspectives on the integration of e-PROMs in routine clinical care. Eur J Oncol Nurs.

[60] Unni E, Coles T, Lavallee DC et al (2023) Patient adherence to patient-reported outcome measure (PROM) completion in clinical care: current understanding and future recommendations. Qual Life Res. 10.1007/s11136-023-03505-y 10.1007/s11136-023-03505-y PMC10784330 37695476

[61] Helleman J, Van Eenennaam R, Kruitwagen ET (2020). Telehealth as part of specialized ALS care: feasibility and user experiences with “ALS home-monitoring and coaching. Amyotroph Lateral Scler Front Degener.

[62] Haulman A, Geronimo A, Chahwala A, Simmons Z (2020). The use of telehealth to enhance care in ALS and other neuromuscular disorders. Muscle Nerve.

[63] Rocque GB, Dent DN, Ingram SA (2022). Adaptation of remote symptom monitoring using electronic patient-reported outcomes for implementation in real-world settings. JCO Oncol Pract.

[64] Aapro M, Bossi P, Dasari A (2020). Digital health for optimal supportive care in oncology: benefits, limits, and future perspectives. Support Care Cancer.

[65] Basch E, Barbera L, Kerrigan CL, Velikova G (2018). Implementa on of patient-reported outcomes in routine. ASCO educational book.

[66] Khatri P, Conaway MR, Johnston KC (2012). Ninety-day outcome rates of a prospective cohort of consecutive patients with mild ischemic stroke. Stroke.

[67] Fischer U, Baumgartner A, Arnold M (2010). What is a minor stroke?. Stroke.

[68] Chirra M, Marsili L, Wattley L (2019). Telemedicine in neurological disorders: opportunities and challenges. Telemed e-Health.

[69] De Farias FACD, Dagostini CM, Bicca YDA (2020). Remote patient monitoring: a systematic review. Telemed e-Health.

[70] Kruklitis R, Miller M, Valeriano L (2022). Applications of remote patient monitoring. Prim Care Clin Off Pract.

[71] Sharrief AZ, Guzik AK, Jones E (2023). Telehealth trials to address health equity in stroke survivors. Stroke.

[72] Schmidt R, Geisler D, Urban D (2023). Stroke survivors’ preferences on assessing patient-reported outcome measures. J Patient-Reported Outcomes.

[73] Oczkowski C, O’Donnell M (2010). Reliability of proxy respondents for patients with stroke: a systematic review. J Stroke Cerebrovasc Dis.

[74] Furnham A (1986). Response bias, social desirability and dissimulation. Pers Individ Dif.

[75] Fisher RJ (1993). Social desirability bias and the validity of indirect questioning. J Consum Res.

